# Microbiome Alteration Prevents Abstinence-Induced Nicotine Withdrawal in a Well-Established Planarian Model

**DOI:** 10.7759/cureus.89075

**Published:** 2025-07-30

**Authors:** Enrique Mentado-Sosa, Juan Miguel Guerra-Solano, Robert B Raffa, Oné R Pagán, John Pisciotta

**Affiliations:** 1 Department of Biology, West Chester University, West Chester, USA; 2 School of Pharmacy, Temple University, Philadelphia, USA

**Keywords:** ampicillin, antibiotic, girardia dorotocephala, kanamycin, microbiome, nicotine withdrawal, planarian, substance use disorder (sud)

## Abstract

The prevalence of substance use disorders (SUDs) is unequally distributed across socioeconomic strata. Although several genetic predispositions and psychosocial influences play integral roles, environmental factors are undoubtedly additional contributors. We propose that a potential common factor could be diet. More specifically, circumstances such as economic challenges could lead to limited food choices and poor-quality diets, and this could result in differences in microbiome composition compared to less SUD-susceptible populations having otherwise similar risk factors. The current study investigated the effect of altering the microbiome on drug withdrawal from nicotine using a standard planarian model. Planarians (*Girardia dorotocephala*) were treated with the broad-spectrum antibiotics ampicillin (a ß-lactam) and kanamycin (a non-ß-lactam), alone and in combination, and microbiomes were analyzed using culture techniques, microscopy, and metagenomic methods. Alphaproteobacteria such as *Sphingomonadaceae* were detected in the microbiome. Ampicillin or kanamycin reduced the microbiome diversity, notably reducing *Sphingomonas* and *Pedobacter* bacteria. One-week treatment with ampicillin and kanamycin did not affect planarian spontaneous locomotor activity. However, pretreatment with ampicillin, but not kanamycin or the combination, significantly attenuated abstinence-induced nicotine withdrawal-like behavior. These results suggest that alteration of the microbiome decreases nicotine withdrawal in this planarian species, and, more broadly, supports the idea that the microbiome might influence the susceptibility and/or maintenance of SUDs.

## Introduction

The use of nicotine products remains a leading cause of preventable morbidity and mortality [1]. For example, smoking carries an almost threefold greater risk of preventable death than never smoking [2]. However, attempts to stop chronic nicotine use produce a well-characterized and defined withdrawal syndrome that drives reinstatement of use (relapse) [3-5]. Unfortunately, use prevalence is higher among lower socioeconomic groups, and their attempts at quitting are less likely to be successful [6]. Therefore, a complete understanding of nicotine withdrawal from all sources possible is needed.

The microbiome plays a critical role by influencing the host's peripheral (PNS) and central (CNS) nervous system homeostatic and stress-related physiological processes. Mutualistic microorganisms within the gastrointestinal tract aid in the digestion of advantageous substances and detoxification of harmful substances [7]. In addition, bacterial populations synthesize B-group vitamins, including niacin, folic acid, and other growth factors such as short-chain fatty acids and host essential amino acids [8,9]. The microbiome also influences host immunity and can affect the CNS, including drug misuse behavior [10]. A relationship between the microbiome and nicotine withdrawal has been shown in humans [11] and mammalian models [12].

Freshwater planarians have been widely studied in regeneration and developmental biology research (e.g., [13-17]). More recently, they have been developed as animal models in pharmacology and toxicology, due to their centralized "brain" (cerebral ganglion) and spinal processes (longitudinal ventral nerve chords), and neurochemical similarity to vertebrates (e.g., [15,16,18-22]). A particular advantage of using planarians as pharmacological models is the range of behavioral responses that these organisms exhibit in response to exposure to - and withdrawal from - abused drugs [20]. For example, planarians display withdrawal-like behaviors in response to nicotine and other drugs (e.g., [23-29]. An extensive number of controls, even including elimination of a potentially confounding effect of osmolarity effects have established that the withdrawal phenomenon in planarians is a robust model of drug withdrawal in humans [16,30]. 

The microbiomes of two planarian species have been characterized previously: *Schmidtea mediterranea* [31] and *Dugesia japonica* [32]. In both species, the dominant members of the bacterial community are the Bacteroidota and Pseudomonadota. It was the purpose of the present study to test the hypothesis that alteration of the microbiome would affect the withdrawal from nicotine in a planarian model of human withdrawal.

A very early version of this article was previously posted to the medRxiv preprint server on April 27, 2020.

## Materials and methods

**Figure 1:**
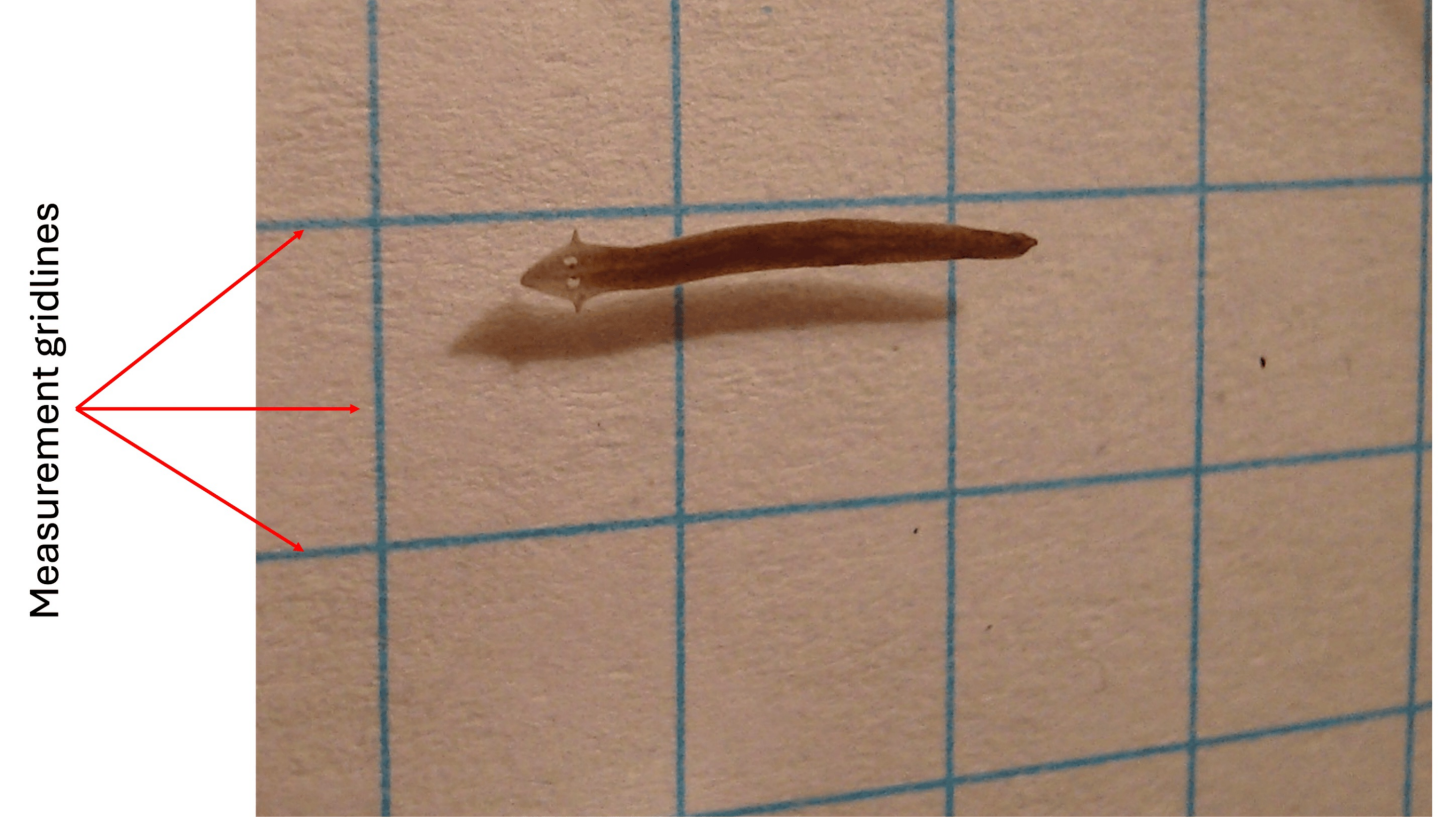
Girardia dorotocephala Original photograph of *G. dorotocephala*, courtesy of the authors.

*G. dorotocephala* and *P. gracilis* planarians (Figure 1) were purchased from Ward’s Science (Rochester, NY; GD#470176-558, PG#470176-452). (-)-Nicotine hydrogen tartrate salt (#N5260), kanamycin, ampicillin sodium salt, and general laboratory chemicals and supplies were purchased from Fisher Scientific (Suwanee, GA), Sigma-Aldrich (St. Louis, MO), or Gold Biotechnology (St. Louis, MO). Graphs and statistical analyses were performed using the Prism software package (GraphPad Inc., San Diego, CA). Upon arrival, planarians were transferred to artificial pond water (APW: NaCl, 6 mM; NaHCO_3_, 0.1 mM; CaCl_2_, 0.6 mM in distilled water) and were allowed to acclimate to laboratory conditions (room temperature in the dark) for at least 24 hours before use. The APW was changed at least once daily except during weekends and always before experiments. All plasticware and glassware were rinsed with APW prior to experiments, and the experiments were conducted at room temperature in APW. All reagents were prepared as APW solutions.

Motility measurements

Planarian motility (spontaneous locomotor activity) was measured using established protocols (e.g., [33], as modified in [34,35]). Briefly, a planarian was individually placed into a 6-cm plastic dish, and the dish was placed on top of a 1-cm^2^ gridline. Five mL of the experimental solution was poured over the planarian, and after one minute, the gridlines crossed by the planarian were counted. As the planarian glided around, the number of times it crossed or recrossed a line was counted each minute over five minutes. The data were analyzed by plotting the cumulative crosses as a function of time, fit to the appropriate linear regression model.

The analysis yielded the gliding velocity of the planarian in cm/minute. We first assessed whether antibiotic exposure would affect the normal motility of planarians. Planarians (N = 8 per group, approximately 1 cm in length, and assigned randomly to groups) were individually acclimated to APW, 1 mg/mL ampicillin, 1 mg/mL kanamycin, or the combination of both antibiotics for one week prior to motility testing. To assess nicotine-induced abstinence withdrawal, planarians (N = 6) were exposed to 50 μM nicotine for one hour under control conditions (APW), and then their motility was tested for five minutes as described above.

Culture-based and microscopic analysis

Following the seven-day antibiotic treatment period, planarians from each group were weighed and then homogenized in APW using sterile, disposable mortar and pestles. The homogenates were serially diluted, then spread-plated onto sterile brain heart infusion (BHI) agar plates, and incubated at room temperature for seven days. After quantifying the number of colonies on each plate, Gram-stained slides of four representative microbes from *G. dorotocephala* and *P. gracilis* planarian BHI plates were generated and visualized using microscopy.

Metagenomic DGGE (denaturing gradient gel electrophoresis) analysis

DNA was extracted from each of the eight worm homogenates using the PowerBioFilm® DNA Isolation Kit (Mo Bio Inc., Carlsbad, CA). The isolated DNA from each worm group was amplified by polymerase chain reaction (PCR) using 16S primers 338f and 907rev. The 16S ribosomal RNA gene was amplified using the forward primer 338_f_GC (5’ - CGC CCG CCG CGC GCG GCG GGC GGG GCG GGG GCA CGG GGG GAC TCC TAC GGG AGG CAG - 3’) (5 μM) and the reverse primer 907r (5’ - CCG TCA ATT CCT TTG AGT TT - 3’) (5 μM) obtained from Integrated DNA Technologies (Coralville, IA). As necessitated for DGGE separation, the forward primer contained a 27-base GC clamp on the 5’ end. Extracted DNA samples were subject to PCR with an initial denaturation step at 94˚C for 10 minutes, followed by 35 cycles of 94˚C (30 s), 53˚C (30 s), and 72˚C (45 s) with final extension at 72˚C for seven minutes. Following amplification, samples were loaded onto a DGGE gel with a 70-40% gradient was run for 16 hours at 90 V, then stained with Sybr-gold for the analysis of differences in microbial communities in the different treatment groups.

16s rDNA library preparation

DNA extracted from each of the worm homogenates was quantified spectrophotometrically and adjusted to 10 ng of genomic DNA. The 16S barcodes for each sample 1-8 were thawed at room temperature, and then each tube was individually mixed. Once vortexed, the 16S barcodes were kept on ice as PCR reactions were set up as follows to create the barcoded library as specified by the manufacturer, Oxford Nanopore Technologies (Oxford, UK).

The eight barcoded samples were amplified using the following PCR conditions: initial denaturation at 95 °C for one minute, followed by 25 cycles, each with 20 seconds of denaturation at 95 °C, annealing at 55 °C for 30 seconds, and extension at 65 °C for two minutes, with one final extension at 65 °C for five minutes. PCR-amplified samples were transferred to fresh tubes, and 30 ul of AMPure XP beads were mixed for five minutes at room temperature. Tubes were centrifuged at 10,000 g for 30 seconds and then placed on a magnet to immobilize beads. Samples were washed with 200 uL of 70% ethanol, then centrifuged for 30 seconds, and placed back onto the magnet. Pellets were air-dried prior to resuspension in 10 uL of 10 mM Tris-HCl pH 8.0, with 50 mM NaCl at room temperature for two minutes. In a single 1.5 ml Eppendorf tube, each of the eight barcoded libraries was pooled in the appropriate ratios to a total of 100 fmoles, and 10 mM Tris-HCl pH 8.0 with 50 mM NaCl was added, reaching a total volume of 10 uL. One (1) uL of the rapid adapter RAP was pooled with the library and mixed, and the reaction was incubated at room temperature for five minutes.

MinION 16s rDNA sequence analysis

The sequencing buffer (SQB), loading beads (LB), flush tether (FLT), and one tube of flush buffer (FB) were thawed at room temperature and then kept on ice. The SQB and FLB tubes were mixed individually by vortexing and spun down. Once mixed, each tube was kept on ice. The MinION nanopore sequencing device was opened, and the flow cell was placed inside and primed as per the manufacturer’s instructions. The SQB and LB were mixed individually by pipetting, and then 70 uL of the pooled library was loaded, and the samples were sequenced using a 1 TB SSD hard drive computer. Once complete, the data were analyzed via the EPI2ME online database.

## Results

Antibiotic treatment did not alter *G. dorotocephala* activity

**Figure 2:**
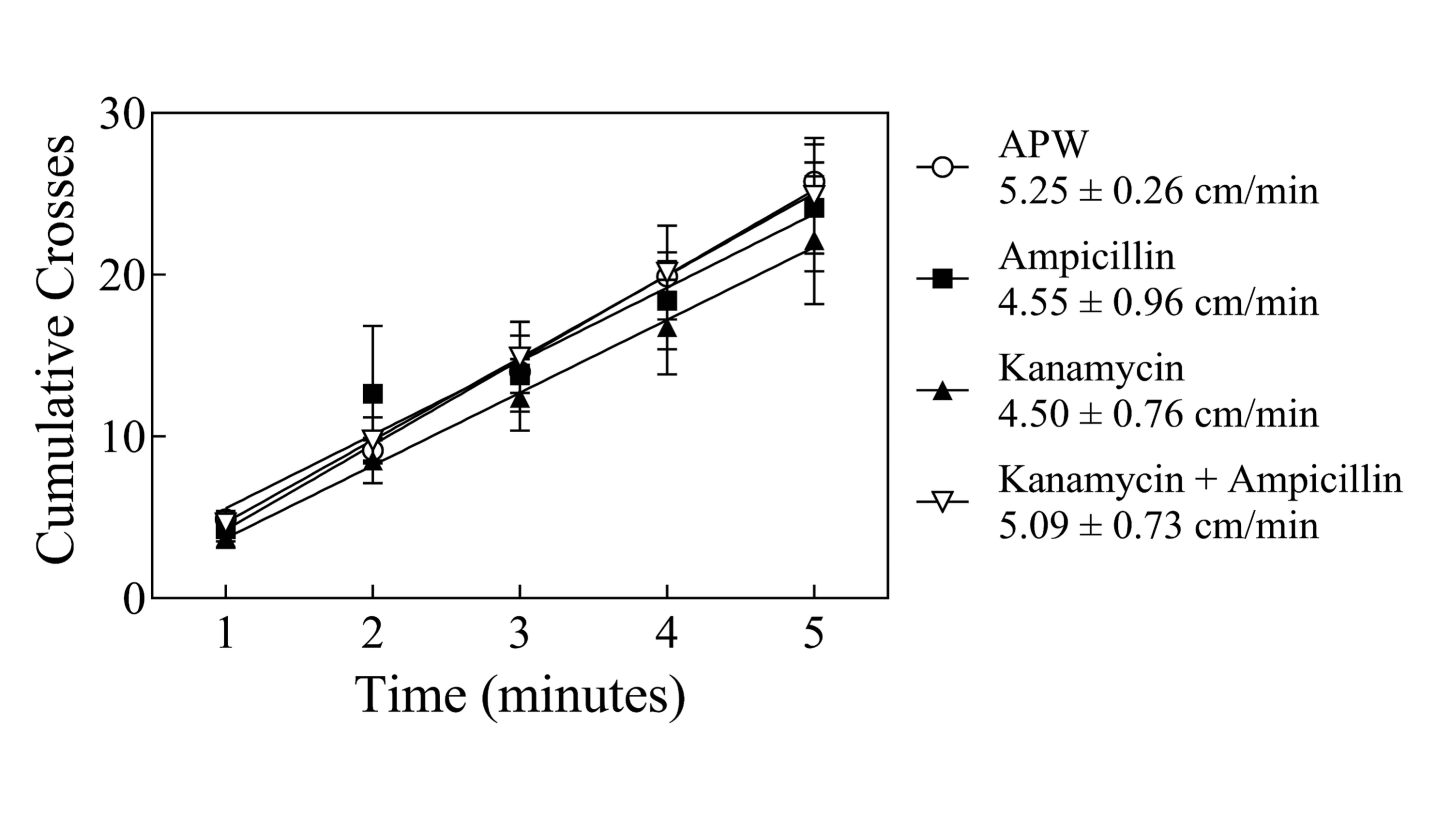
No effect of antibiotic treatment on spontaneous activity Lack of effect of antibiotic exposure on *G. dorotocephala* spontaneous activity in artificial pond water (APW) compared to non-treated controls. Each line represents the velocity of a mean of 8 planarians ± SEM, as indicated in the figure. There was no difference (P > 0.05) among the four conditions tested.

There was no significant difference (P > 0.05) in the velocity of on *G. dorotocephala* planarians tested without antibiotic treatment compared to treatment with 1 mg/mL ampicillin alone, 1 mg/mL kanamycin alone, or the combination of ampicillin plus kanamycin, both at 1 mg/mL (Figure 2, as indicated). Based on the absence of effect on this normal spontaneous behavior, testing for an effect on nicotine withdrawal was pursued.

Antibiotic treatment alters the planarian microbiome

**Figure 3:**
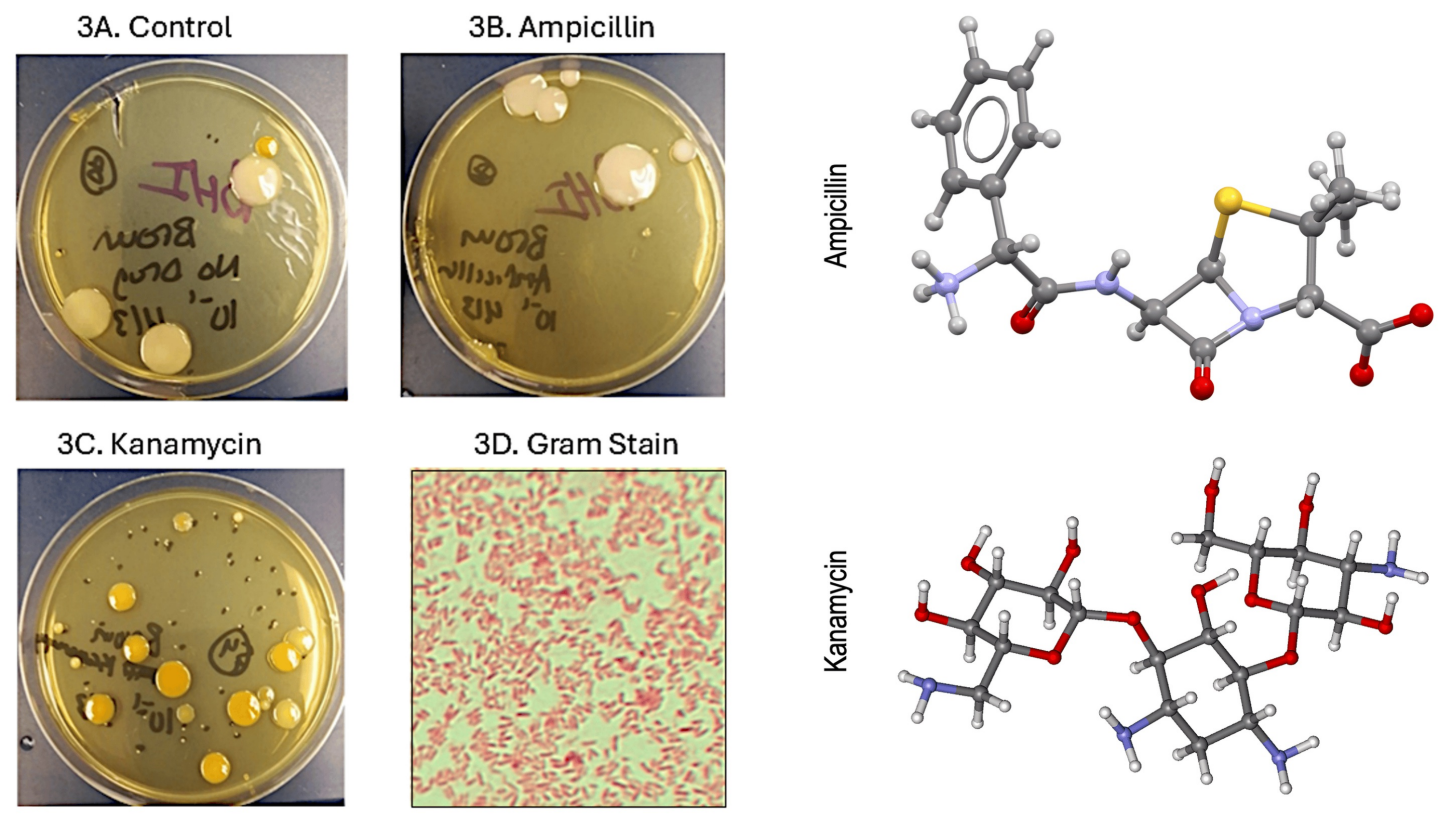
Effect of antibiotic treatment on G. dorotocephala microbiome. Antibiotic treatment appears to differentially alter the *G. dorotocephala* microbiome depending on drug class. Treatment with the aminoglycoside kanamycin (3C) inhibited the large salmon-pigmented colonies present in the no drug control (3A) that were evidently resistant to the ß-lactam antibiotic ampicillin (3C).  Kanamycin treatment resulted in an increase in small colonies as well as flat, yellow-pigmented colonies (3B). Gram staining of randomly selected yellow colony revealed gram negative bacilli (3D). We did not confirm the identities of individual colonies. However, the colony morphology, yellow pigmentation, and presence of gram-negative rods is consistent with *Sphingomonas*, which was detected metagenomically.

**Figure 4:**
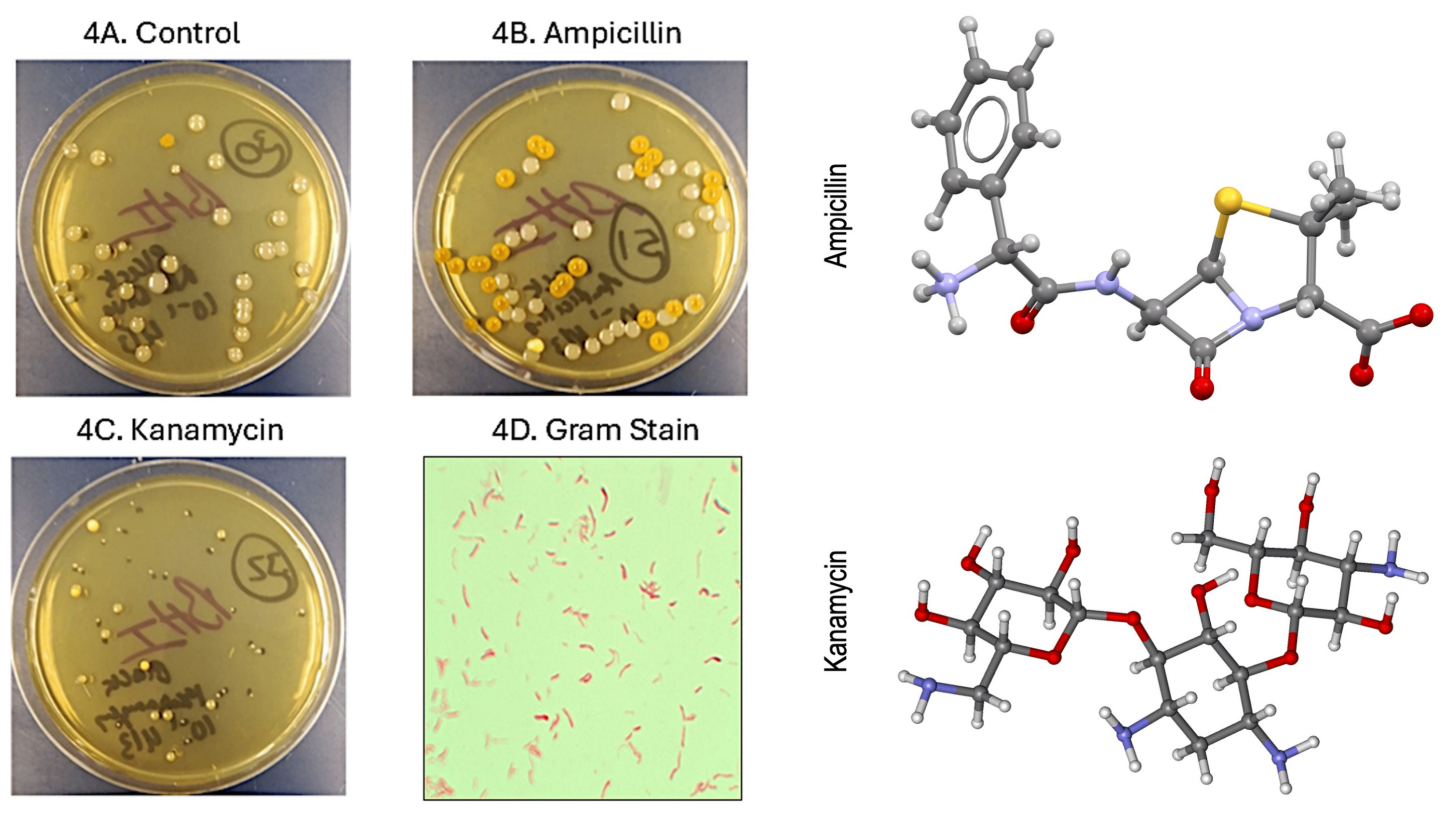
Effect of antibiotic treatment on P. gracilis microbiome. Antibiotic treatment differentially alters the *P. gracilis* black planarian microbiome depending on drug class. Ampicillin did not substantially increase the large light pink colonies but caused an evident enrichment in yellow-pigmented colonies (4B) compared to the no drug control (4A). In contrast, kanamycin inhibited large colonies and selected for smaller colonies (4C).  Gram staining of a selected yellow-pigmented colony revealed gram negative bacilli (4D).

Treatment with two broad-spectrum antibiotics, ampicillin and or kanamycin, altered the microbiome of planarians, as determined by culture-based and metagenomic methods. Following a seven-day course of antibiotic treatment, diluted worm homogenates resulted in the disparate growth of various bacterial colony types on BHI agar. As seen in Figure 3, the untreated control *G. dorotocephala* contained large, light pink (i.e., salmon-colored) colonies along with a yellow pigmented isolate. Ampicillin treatment did not inhibit the salmon-colored colonies. Kanamycin inhibited the salmon-colored colonies but not the yellow pigmented colonies. A similar effect was noted with black planarian *P. gracilis* (Figure 4).

**Figure 5:**
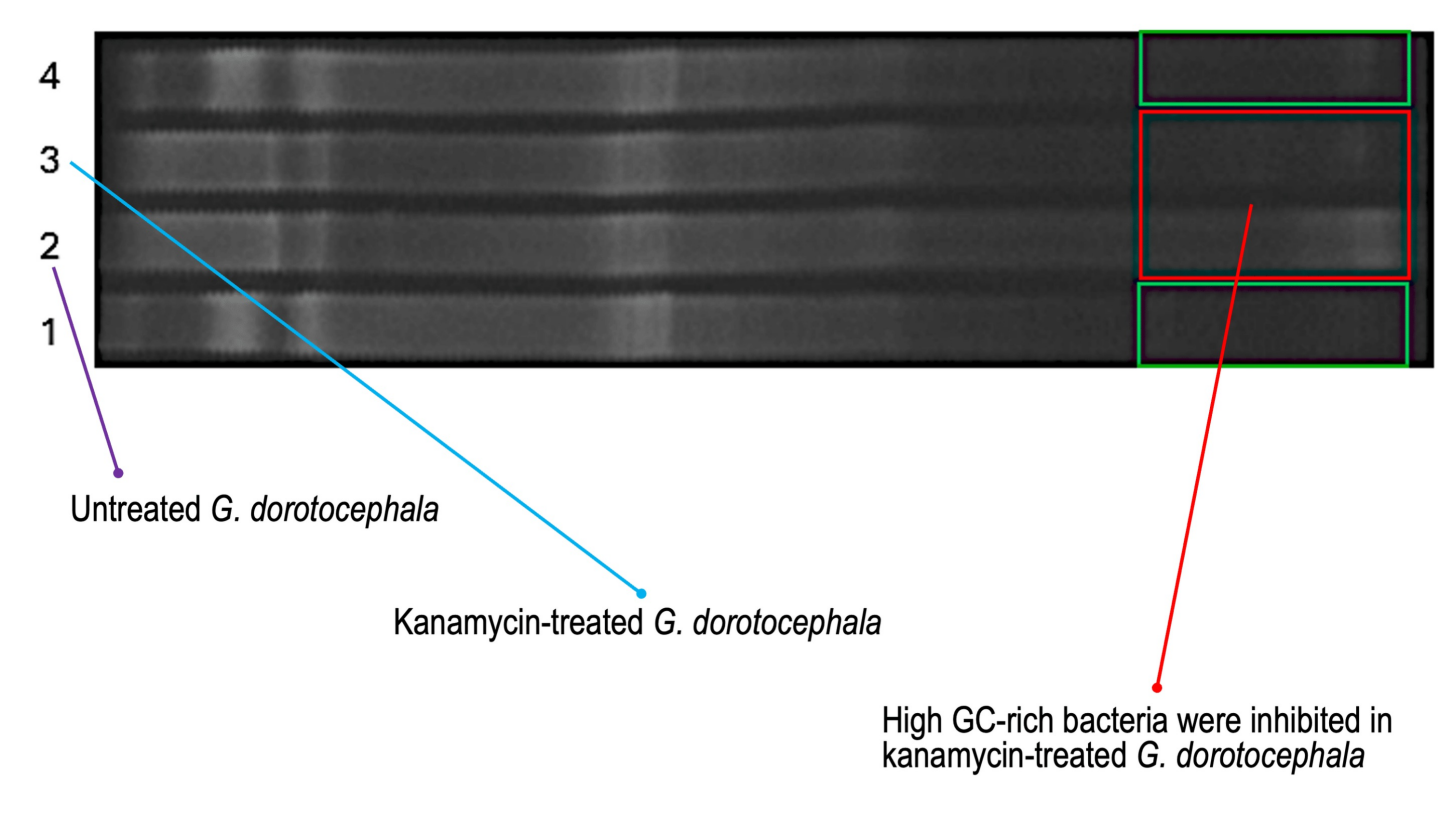
Metagenomic DGGE community analysis of brown and black planarians following treatment with kanamycin. The denaturing gradient gel electrophoresis (DGGE) gel contains a gradient of DNA denaturant (urea) that increases from left to right such that AT-rich sequences denature first and separate nearer the left, while GC-rich sequences progress toward the right. DGGE analysis of 16S rDNA indicates apparent differences in the microbiome community composition between untreated *G. dorotocephala* brown planaria (lane 2) and untreated *P. gracilis* black planaria (lane 4). The red box suggests that high GC-containing bacteria were inhibited by kanamycin in *G. dorotocephala* (lane 3) compared to untreated *G. dorotocephala* (lane 2). Green boxes suggest that kanamycin induced a similar inhibitory effect on GC-rich bacteria of *P. gracilis* near the right part of the gel (lane 1) as compared to lane 4. However, near the left part of the gel, kanamycin also partly inhibited a band of AT-rich bacteria in only the black planaria, *P. gracilis*, as indicated by comparing lanes 1 and 4.

**Figure 6:**
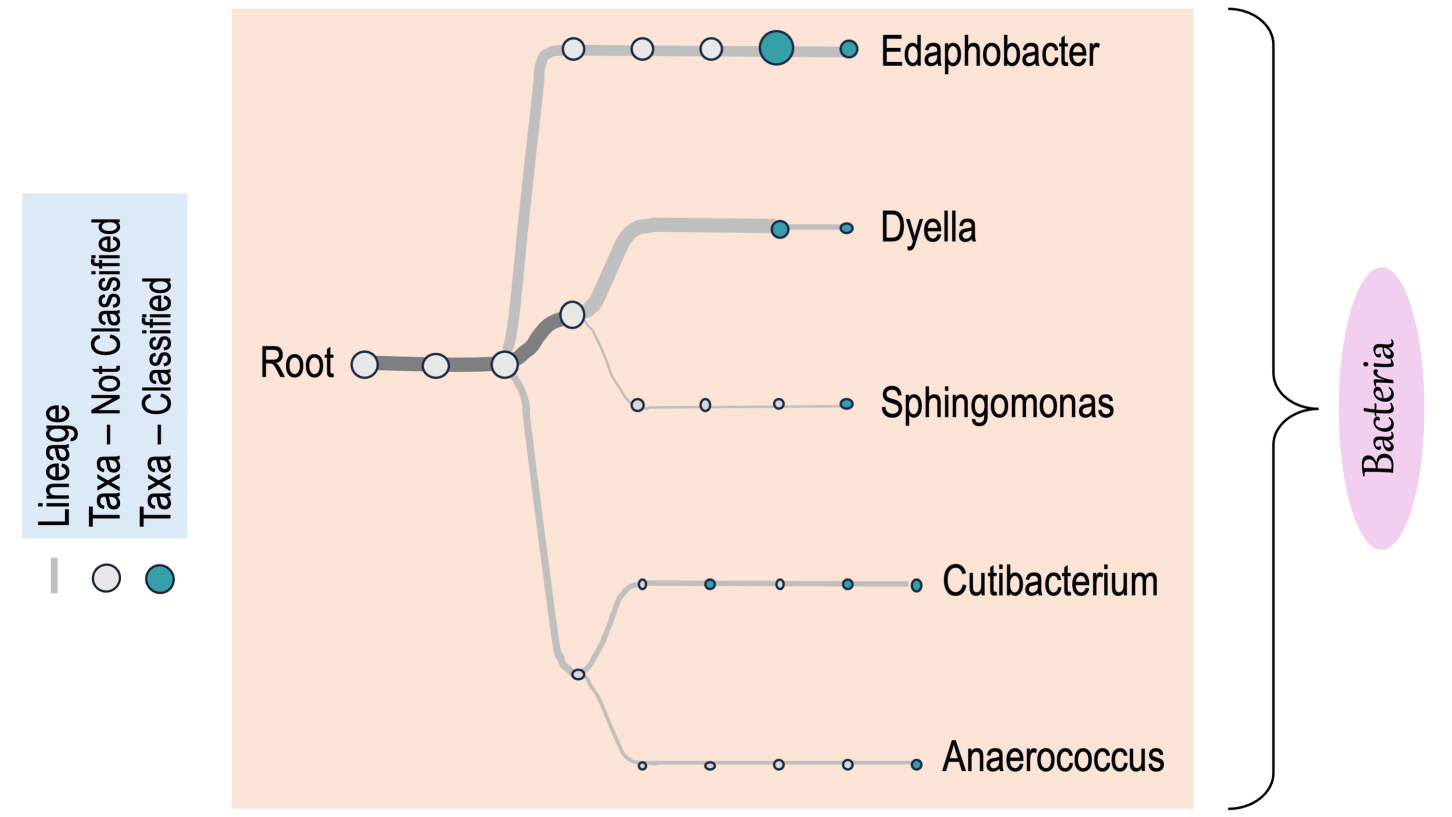
NCBI taxonomy tree of untreated brown planaria. Similar to brown worms, untreated black worms contained detectable *Sphingomonas* and *Pedobacter*, suggesting that the microbiomes of disparate genera of planarians overlap. No reads were detected by MinION for the ampicillin-treated black worms, whereas kanamycin-treated black worms indicated *Pedobacter*, *Sphingomonas*, and *Mucilaginbacter*. No amplicon reads were detected by the MinION for the ampicillin- and kanamycin-treated black worms. *Pedobacter *was previously detected in the European planarian *Schmidtea mediterranea* [36,37].

Metagenomic methods likewise indicated that antibiotic treatment perturbs the microbiome of planaria. DGGE analysis of brown and black planaria treated with kanamycin indicated that the bacterial community composition was most heavily reduced in the abundance of GC-rich bacteria, as seen by the reduction in bands lower on the gel (Figure 5). Sequence analysis of the 16s rRNA gene further indicated antibiotic-mediated alteration of bacterial populations in the drug-treated groups as compared to the untreated control planaria. More specifically, 16S rDNA gene sequence analysis of planarian barcoded libraries yielded 189 total reads. The most common bacterial taxa identified in the untreated control *G. dorotocephala* were *Edaphobacter*,* Dyella*, *Sphingomonadaceae*, and *Cutibacterium* (Figure 6). For ampicillin-treated brown worms, only *Sphingomonadaceae*, which are innately beta-lactam resistant, were detected. Kanamycin-treated brown worms contained *Streptococcus* and *Pedobacter*. Ampicillin plus kanamycin-treated brown worms contained *Acidobacteriaceae*, *Rhodanobacteraceae*, and *Oxalobacteraceae*. Therefore, different antibiotic treatments alter the composition of the *G. dorotocephala* bacterial microbiome

*P. gracilis* controls contained *Sphingomonas* and *Pedobacter*, similar to *G. dorotocephala*. No reads were detected for the ampicillin-treated black worms. Kanamycin-treated black worms indicated *Pedobacter*, *Sphingomonas*, and *Mucilaginbacter*. No amplicon reads were detected by the MinION for the ampicillin plus kanamycin-treated black planarian, *P. gracilis*.

Ampicillin, but not ampicillin plus kanamycin or kanamycin alone, attenuated abstinence-induced nicotine (50 µM) withdrawal in *G. dorotocephala*

**Figure 7:**
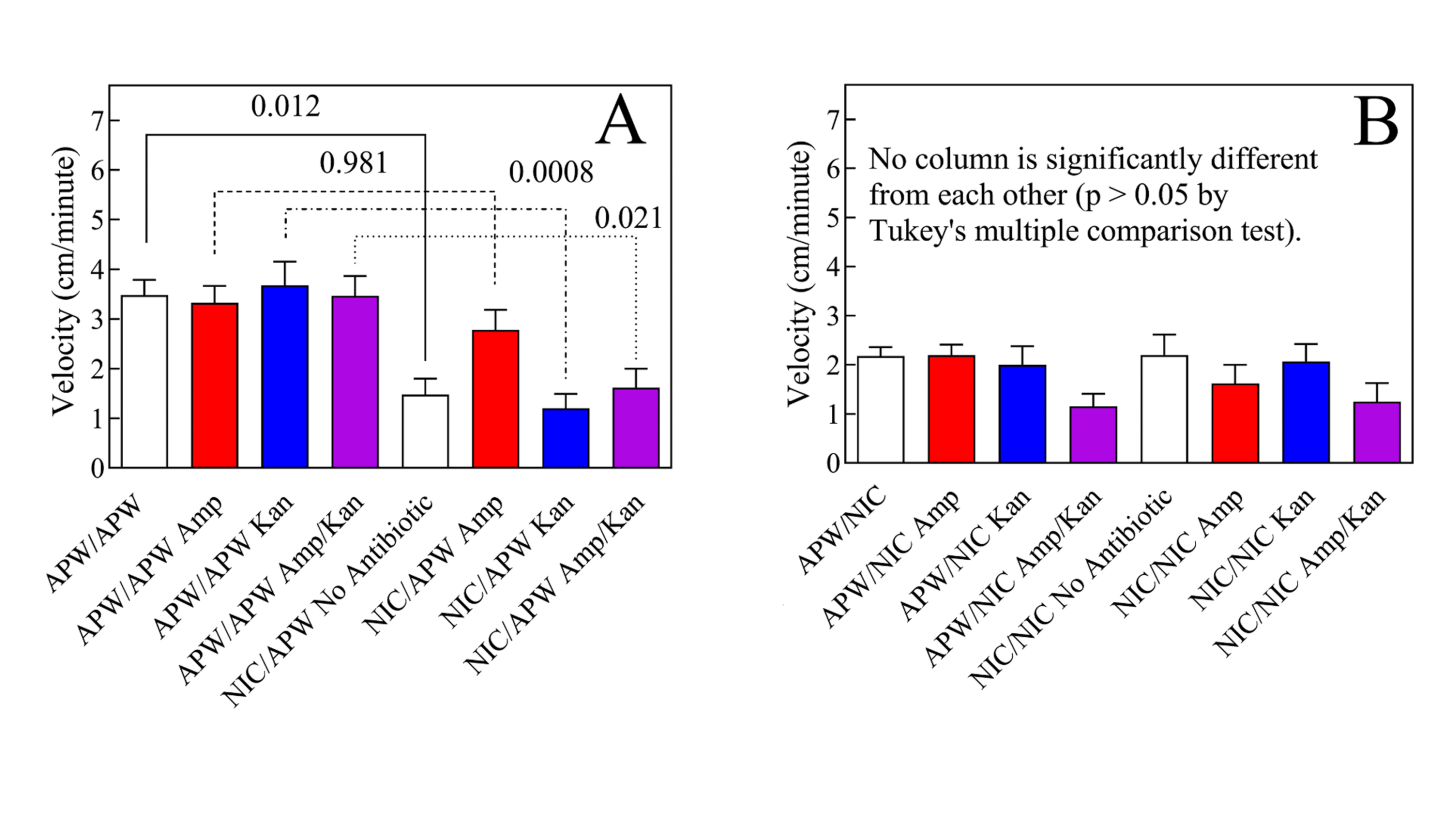
Ampicillin significantly attenuated 50 μM nicotine abstinence-induced withdrawal The worms pre-exposed to the antibiotics for seven days were washed with APW and then exposed to either APW or nicotine for one hour. These worms were then washed again with APW and “post-exposed” to either nicotine or APW and their five-minute motility was measured as described while they were in the “post” solution. (A) Exposure to ampicillin (1 mg/mL), kanamycin (1 mg/mL), or both ampicillin and kanamycin, both at 1 mg/mL has no effect on planarian velocity, consistent with the results shown in Figure 1. Pre-exposure to 50 μM nicotine followed by APW wash induces abstinence-induced withdrawal with is alleviated by pre-exposure to ampicillin, but not kanamycin or the combination of both (see text). (B) 50 μM nicotine decreases planarian velocity regardless of the time of application (see text). Multiple comparisons via Tukey’s test. Each bar represents the average of six worms; the error bars represent the SEM.

As shown in Figure 7A, planarians that were pretreated with APW in the absence of either antibiotic and then tested in APW displayed a motility velocity of 3.48 ± 0.76 cm/min. Planarians that were exposed to ampicillin in APW and then tested in APW displayed a motility velocity of 3.33 ± 0.82 cm/min. Planarians that were exposed to kanamycin in APW and then tested in APW displayed a motility velocity of 3.68 ± 1.17 cm/min. Planarians that were exposed to ampicillin plus kanamycin in APW and then tested in APW displayed a motility velocity of 3.47 ± 0.97 cm/min. There was no significant difference (p > 0.05) among these groups. Planarians that were not exposed to either antibiotic, pretreated with nicotine, and then tested in APW, displayed a motility velocity of 1.48 ± 0.79 cm/min, which was significantly different (p = 0.012) from planarians that were pretreated in APW and then tested in APW in the absence of either antibiotic. Such a decrease in locomotor velocity has been interpreted as a sign of abstinence-induced withdrawal in planaria with a variety of substances [38]. Planarians that were exposed to ampicillin, treated with nicotine, and then tested in APW displayed a motility velocity of 2.78 ± 1.00 cm/min, which was not significantly different (p = 0.981) from ampicillin-exposed planarians that were pretreated in APW and then tested in APW, suggesting that ampicillin has an alleviating effect on nicotine’s abstinence-induced withdrawal. Planarians that were exposed to kanamycin, treated in nicotine, and then tested in APW displayed a motility velocity of 1.20 ± 0.72 cm/min, which was significantly different (p = 0.0008) from planarians that were pretreated with kanamycin in APW and then tested in APW, showing that kanamycin had no effect on the aforementioned nicotine effect, as were planarians that were pretreated with ampicillin plus kanamycin, treated with nicotine and then tested in APW, which displayed a motility velocity of 1.62 ± 0.92 cm/min, which was significantly different (p = 0.021) from planarians that were pretreated in APW and then tested in APW.

As shown in Figure 7B, there was no significant difference (p > 0.05) between the following groups: planarians that were pretreated in APW and then tested in nicotine in the absence of antibiotic pre-exposure (2.18 ± 0.44 cm/min), planarians that were pretreated with ampicillin and then tested in nicotine (2.20 ± 0.52 cm/min), planarians that were pretreated with kanamycin and then tested in nicotine (2.00 ± 0.92 cm/min), planarians that were pretreated with ampicillin plus kanamycin and then tested in nicotine (1.15 ± 0.63 cm/min), planarians that were pretreated in nicotine and then tested in nicotine in the absence of antibiotic (2.20 ± 1.01 cm/min), planarians that were exposed to ampicillin, treated with nicotine and then tested in nicotine (1.62 ± 0.93 cm/min), planarians that were exposed to kanamycin, treated with nicotine and then tested in nicotine (2.07 ± 0.86 cm/min), and planarians that were exposed to both ampicillin and kanamycin, treated with nicotine, and then tested in nicotine (1.25 ± 0.92 cm/min).

## Discussion

The antibiotics ampicillin and kanamycin, either independently or in combination, had no effect on normal planarian activity. Therefore, we continued to investigate the potential effect of microbiome alteration by the two antibiotics ampicillin (a ß-lactam) and kanamycin (a non-ß-lactam). Metagenomic analysis indicated that antibiotic treatment reduced the diversity and quantity of detectable bacterial populations in treated planarians. We therefore proceeded to test the potential effect of microbiome alteration on abstinence-induced nicotine withdrawal in an established planarian model [16,20,30].

Planarians that were pretreated in APW and then tested in APW without ampicillin displayed a normal motility velocity, a control that indicates that there was no change in normal activity over the observation period. Planarians that were pretreated in APW and then tested in APW with kanamycin displayed a normal motility velocity, a control that indicates that kanamycin did not alter normal activity over the observation period. Planarians that were pretreated in APW and then tested in APW with kanamycin displayed a normal motility velocity, a control that indicates that kanamycin did not alter normal activity over the observation period. Planarians that were pretreated in APW and then tested in APW containing ampicillin plus kanamycin displayed normal motility velocity, a control that indicates that a combination of ampicillin plus kanamycin did not alter normal activity over the observation period. There was no significant difference among these control groups, so we proceeded. Planarians that were pretreated with nicotine and then tested in APW without antibiotics displayed a significant reduction in motility, a finding that extensive previous work has demonstrated is a rigorous model of abstinence-induced withdrawal in the planarian model [16,20,30]. Planarians that were pretreated with nicotine and then tested in APW containing ampicillin displayed a motility velocity not significantly different from planarians pretreated in APW and then tested in APW containing ampicillin, demonstrating that ampicillin prevented abstinence-induced nicotine withdrawal. Planarians that were pretreated with nicotine and then tested in APW containing kanamycin displayed significantly less motility than planarians that were pretreated in APW and then tested in APW containing kanamycin, showing that kanamycin did not prevent abstinence-induced nicotine withdrawal. Planarians that were pretreated with nicotine and then tested in APW containing ampicillin plus kanamycin displayed significantly less motility than planarians that were pretreated in APW and then tested in APW containing ampicillin plus kanamycin, showing that the combination of ampicillin plus kanamycin did not prevent abstinence-induced nicotine withdrawal.

Taken together, the results indicate that a ß-lactam broad-spectrum antibiotic (ampicillin) prevents abstinence-induced withdrawal from nicotine, but a non-ß-lactam broad-spectrum antibiotic (kanamycin) does not. It is unclear why the combination did not have an effect. One possibility is that the combination effectively gave the worms a double dose of antibiotic, effectively antagonizing ampicillin’s effect. Note that this does not reflect on clinical activity; it only reports the findings of nicotine withdrawal using the protocol of this test system.

In retrospect, there is a possible prior indication in the literature that antibiotics can affect the withdrawal behavior in planarians. Specifically, Rawls et al. [38] reported that the antibiotic ceftriaxone inhibits physical dependence and abstinence-induced withdrawal from cocaine, amphetamine, methamphetamine, and clorazepate in planarians. However, those authors attributed ceftriaxone’s effect to its interaction with certain glutamate transporter subtypes. In light of the current study, their results might also be interpreted, at least in part, as the effects of ceftriaxone on the planarian microbiome. 

The current study has limitations. Although we attribute the effect of ampicillin on alleviating nicotine withdrawal to changes in the microbiome, it has not conclusively been proven. Further study is required to determine if changes in microbiome alone, transporters, or some combination of the two result in behavioral changes after only one hour versus a week of exposure. Other limitations include small sample sizes, potential confounding effects, and follow-up that specific bands of DNA are GC-rich with molecular evidence (e.g., sequencing or composition analysis). Hopefully, future work will address these gaps to strengthen generalizability.

## Conclusions

This study aimed to determine if microbiome alteration using antibiotics modulates nicotine withdrawal behavior in planarians. The study characterized the microbiome of the widely used model planarian *G. dorotocephala*. Metagenomic results demonstrated that seven-day treatment with the broad-spectrum antibiotics ampicillin or kanamycin reduced the overall diversity of bacteria in the planarian microbiome. Treatment with ampicillin or kanamycin, or the combination of both, had no significant effect on normal planarian motility. Treatment of *G. dorotocephala* for one week with the ß-lactam antibiotic ampicillin alone prevented abstinence-induced nicotine withdrawal in this well-characterized and well-controlled planarian model. Collectively, these results suggest that alteration of the microbiome in planarians affects an aspect of the SUD phenomenon (withdrawal), and, more broadly, that pharmacological modulation of the animal microbiome might offer novel insight into the environmental contributors/influences on the initiation and/or maintenance of substance abuse problems.
