# Correction: Microbiome Alteration Prevents Abstinence-Induced Nicotine Withdrawal in a Well-Established Planarian Model

**DOI:** 10.7759/cureus.c234

**Published:** 2025-08-08

**Authors:** Enrique Mentado-Sosa, Juan Miguel Guerra-Solano, Robert B Raffa, Oné R Pagán, John Pisciotta

**Affiliations:** 1 Department of Biology, West Chester University, West Chester, USA; 2 School of Pharmacy, Temple University, Philadelphia, USA

The original version of the conflict of interest disclosure statement stated the following regarding payments and services: “Western Carolina University's (WCU) Department of Biology and College of Science and Mathematics provided funding for this study, in the form of an RIMS (Research Information Management System) grant to O.R.P. (Oman Research Portal).”

This has been corrected to accurately state the following: “West Chester University’s (WCU) Department of Biology and College of Science and Mathematics provided funding for this study, in the form of an RIMS (Research in Mathematics and Sciences) grant to O.R.P. (Oné R. Pagán).”

